# Chemical Composition and Antibacterial, Antioxidant, and Cytotoxic Activities of Essential Oils from Leaves and Stems of *Aeschynomene indica* L.

**DOI:** 10.3390/molecules29153552

**Published:** 2024-07-28

**Authors:** Linjie Feng, Fan Xu, Shu Qiu, Chengqi Sun, Pengxiang Lai

**Affiliations:** 1 Sdu-Anu Joint Science College, Shandong University, Weihai 264209, China; 202100700236@mail.sdu.edu.cn (L.F.);; 2 Marine College, Shandong University, Weihai 264209, China

**Keywords:** *Aeschynomene indica*, essential oil, antibacterial, synergistic, antioxidant, cytotoxic

## Abstract

The objective of this study was to analyze the chemical composition and evaluate the biological capabilities of the essential oils (EOs) extracted from leaves and stems of wild *Aeschynomene indica* L. plants by the hydrodistillation method. By using GC-FID/MS, fifty-six and fifty-five compounds, representing 95.1 and 97.6% of the essential oils in the leaves and stems, respectively, were characterized. The predominant constituents of *A. indica* EOs were (*E*)-caryophyllene, linalool, viridiflorol, phytol, hexadecanoic acid, trans-verbenol, and α-guaiene. The antibacterial and synergistic activities of the EOs were assessed by microdilution and checkerboard assays. The results revealed a potent inhibition and bactericidal activity against *Staphylococcus aureus* and *Bacillus subtilis* with MICs of 0.312–0.625 mg/mL. When combined with traditional antibiotics, the essential oils of *A. indica* possessed excellent synergistic effects against all tested bacteria. Additionally, the EOs of *A. indica* leaves showed higher antioxidant activity (IC_50_ = 0.11 ± 0.01 µg/mL) compared to the stem oil (IC_50_ = 0.19 ± 0.01 µg/mL) using the ABTS radical scavenging assay. The in vitro cytotoxicity of EOs against human cancer cell lines HepG2, MCF-7, A-549, and HCT-116 was examined, and MTT assays showed that the EOs possessed a significant cytotoxic potential against MCF-7 breast cancer cells, with IC_50_ values of 10.04 ± 1.82 and 15.89 ± 1.66 μg/mL, and a moderate cytotoxic activity against other tested cells. In conclusion, the *A. indica* EOs could be considered a potential source of pharmacologically active compounds.

## 1. Introduction

Plants are susceptible to invasion by numerous infections that may affect different parts of the plant. In order to restrict pathogen attacks, plants produce a variety of secondary metabolites to inhibit the growth of pathogens, such as terpenoids, phenolics, and nitrogen compounds [1]. Essential oils are a complex mixture of several lipophilic and highly volatile constituents that are produced by specific secretory tissues of the plant’s flowers, seeds, leaves, wood, and bark as secondary metabolites. These constituents exhibit an extensive range of pharmacological properties, such as analgesic, antioxidant, antimicrobial, anti-inflammatory, anticancer, anti-virus, neuroprotective, and anti-diabetic effects [2,3]. Since ancient times, medicinal and aromatic plants have been utilized for their wide variety of physiologically active secondary metabolites [4]. The growing interest in pharmacologically potent plant-based natural compounds as alternative therapeutics for treating infections has heightened the attention of scientists worldwide [4]. Essential oils could be interesting candidates for alternative antibiotics since studies have shown that specific essential oils and their isolated components exhibit antibacterial effects against a wide spectrum of pathogens [5]. Furthermore, the utilization of essential oils in combination therapy with traditional antibacterial drugs has demonstrated efficacy in reducing the emergence of resistant strains [6]. When these interactions lead to synergistic effects, they can also be used to enhance the efficiency of therapy. Another advantage of such combinations is the utilization of lower doses, resulting in a decrease in side effects and treatment expenses [7]. Therefore, essential oils are being increasingly investigated as a substitute for reducing the side effects of traditional treatments.

The genus *Aeschynomene* belongs to the Leguminosae family and comprises about 250 species. Plants belonging to the genus *Aeschynomene* have been found to possess hepatoprotective [8], antioxidant [9], antimicrobial [10], anti-inflammatory [11], and anthelmintic properties [12]. In preliminary phytochemical studies of different *Aeschynomene* species, the presence of flavonoid glycosides, pterocarpans, saponins, and chalcones was identified [13,14]. In previous studies, the compounds from *Aeschynomene fascicularis* were examined and demonstrated selective cytotoxic and antiproliferative activities [13]. An extract from the root bark of *A. fascicularis* was found to have a pronounced cytotoxic effect on KB and Hela cells [15]. Moreover, in another research, the extracts of *A. fascicularis* also showed effective cytotoxic activity against DU-145, KB, and Hep-2 cell lines and antiproliferative activity against the KB cell line [16]. 

*Aeschynomene indica* L., an annual herb of the genus *Aeschynomene*, is native to tropical Asia, North Korea, Africa, Oceania, Japan, and China [17,18]. The aerial parts of *Aeschynomene indica* L. have been utilized in Chinese folk medicine to cure a variety of conditions, including urticaria, furuncle, nyctalopia, hepatitis, enteritis, and dysentery [18,19]. Previous research demonstrated that the leaves of *A. indica* and its active ingredient efficiently enhanced wound healing and reduced scar formation. This effectiveness may be attributed to their ability to accelerate the transition of macrophage phenotypes and suppress the expression of TGF-*β*1 and *α*-SMA [19]. Additionally, preliminary phytochemical screening has identified flavonoid glycosides, phenols, and anthocyanidins in *A. indica* [18].

To our knowledge, no research has been conducted on the chemical composition and biological effects of the essential oils extracted from different parts of *Aeschynomene indica* L. This study is the first one to report the chemical composition and antibacterial, antioxidant, and cytotoxic activities of the essential oils obtained from leaves (LEOs) and stems (SEOs) of *A. indica*.

## 2. Results

### 2.1. Chemical Composition

The leaves and stems of *A. indica* were broken into fragments and hydrodistilled to give leaf essential oils (LEOs) and stem oils (SEOs) in yields of 0.12% and 0.07% from air-dried parts of the leaves and stems of *A. indica*, respectively. Table 1 displays the discovered volatiles in the LEOs and SEOs of *A. indica*, together with the percentages of their contents, their calculated retention indices, and their retention indices from the literature. The qualitative analysis of EOs by GC-FID/MS revealed fifty-six and fifty-five compounds, representing 95.1% and 97.6% of the total essential oils in leaf and stem samples, respectively.

Oxygenated monoterpenes (21.0% and 32.9%), sesquiterpene hydrocarbons (34.4% and 20.4%), and oxygenated sesquiterpenes (16.2% and 18.6%) were dominant in the leaf and stem essential oils, respectively. It is widely known that the promising potential of EOs is due to the presence of valuable active ingredients. (*E*)-caryophyllene was the most abundant compound among all constituents, by 17.3% and 10.8% in LEOs and SEOs, respectively. The main constituents of the LEOs were (*E*)-caryophyllene (17.3%), viridiflorol (8.1%), phytol (5.2%), trans-verbenol (4.9%), *α*-guaiene (4.7%), linalool (4.1%), n-octadecanol (3.5%), and pentadecanal (3.4%). In the stem essential oils, the main identified constituents were linalool (11.8%), (*E*)-caryophyllene (10.8%), viridiflorol (9.5%), phytol (7.0%), hexadecanoic acid (6.3%), (*E*)-*β*-ionone (3.3%), *α*-terpineol (3.1%), and *α*-guaiene (3.1%).

### 2.2. Antibacterial Activity

Two Gram-positive and two Gram-negative bacteria were tested for the antibacterial properties of LEOs and SEOs extracted from *A. indica* as well as the standard antibiotic chloramphenicol. The recorded minimum inhibitory concentrations (MICs) and minimum bactericidal concentrations (MBCs) are presented in Table 2. For most of the tested bacterial strains, the bactericidal activities of the LEOs and SEOs showed no noticeable differences in MIC and MBC values, which ranged from 0.312 mg/mL to 2.500 mg/mL. Meanwhile, the test results showed that the *A. indica* EOs possessed moderate antibacterial activity against Gram-positive bacteria *Staphylococcus aureus* and *Bacillus subtilis*, with MICs and MBCs ranging from 0.312 to 0.625 mg/mL, while the EOs showed weak antibacterial activity against Gram-negative bacteria tested (MICs = 1.250 mg/mL and MBCs = 2.500 mg/mL). Both EOs exhibited weaker antibacterial activities compared to the positive control chloramphenicol.

### 2.3. Association Test

The synergistic effect between the *A. indica* EOs and conventional antibiotic agents (streptomycin and chloramphenicol) was investigated by testing the MIC values individually and in combination using a checkerboard assay [20]. The values of the fractional inhibitor concentration index (FICI) that were determined based on the results of the checkerboard assays are summarized in Table 3 and Table 4, respectively. The synergistic effect of both EOs with antibiotics resulted in a total synergistic effect against all tested bacterial strains (FICI ≤ 0.50), and the reduction of antibiotics MICs ranged from 4- to 16-fold when combined with *A. indica* EOs. Moreover, the best synergistic effect was recorded in the combination of LEOs and streptomycin against *Staphylococcus aureus* and *Pseudomonas aeruginosa*, with FICI values of 0.10 and 0.13, respectively, and the MIC values of streptomycin were reduced 16-fold.

### 2.4. Antioxidant Activity

Antioxidation is a complicated process that typically occurs in multiple mechanisms, and its assessment is commonly conducted through several test methods [21]. The ferric reducing power assay, ABTS cation, and DPPH radical scavenging assay were used to assess the antioxidant properties of the essential oils derived from *A. indica*. Trolox and butylated hydroxytoluene (BHT) served as positive controls. In the radical scavenging assay, leaf essential oils were more active, with IC_50_ values of 1.35 ± 0.06 and 0.11 ± 0.01 mg/mL, followed by stem essential oils, with IC_50_ values of 1.66 ± 0.05 and 0.19 ± 0.01 mg/mL (Table 5). Trolox equivalents (TEs) in the FRAP system were shown by the leaf essential oils of 103.78 ± 10.23 μmol Trolox × g^−1^, followed by stem essential oils of 57.75 ± 7.63 μmol Trolox × g^−1^. Thus, leaf essential oils demonstrated greater potential for antioxidants than plant stem essential oils. *A. indica* essential oils demonstrated considerably lower radical scavenging power and reducing ability compared to the positive control. Neither of the two oils was as effective as the reference antioxidants BHT and Trolox. These findings suggested that *A. indica* essential oils displayed moderate antioxidant effects.

### 2.5. Cytotoxic Activity

**Figure 1:**
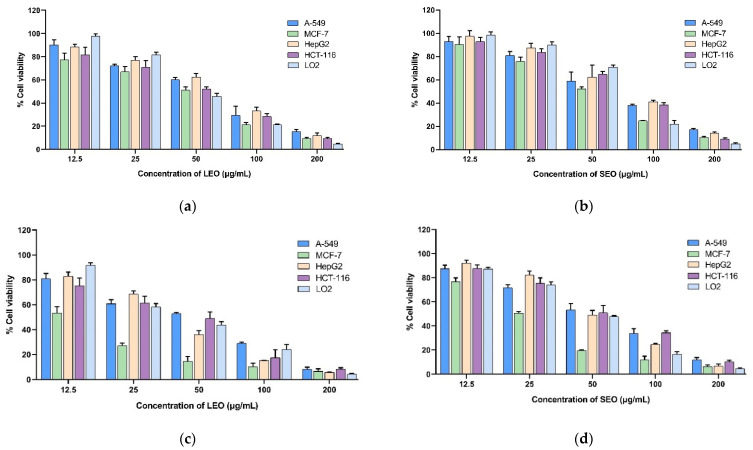

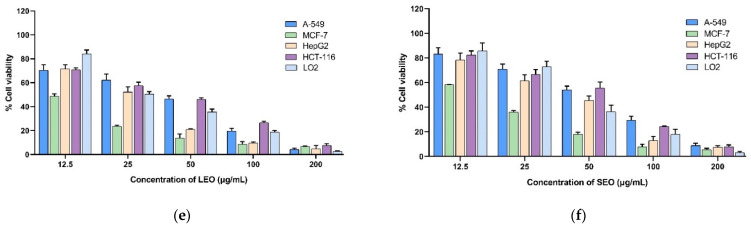
(**a**) Cytotoxic activity of LEOs for 24 h; (**b**) cytotoxic activity of SEOs for 24 h; (**c**) cytotoxic activity of LEOs for 48 h; (**d**) cytotoxic activity of SEOs for 48 h; (**e**) cytotoxic activity of LEOs for 72 h; and (**f**) cytotoxic activity of SEOs for 72 h. Doxorubicin was used as a positive control. IC_50_: concentration reducing cell growth by 50%.

Four human cancer cell lines (HepG2, MCF-7, A-549, and HCT-116) and a human normal hepatocyte (LO2) cell line were subjected to an MTT cell viability experiment in order to assess the cytotoxic effects of extracted LEOs and SEOs. Doxorubicin was tested as a reference. The results obtained with various doses of *A. indica* EOs extracted from two different organs after 24 h, 48 h, and 72 h of exposure are presented in Figure 1, and the IC_50_ values are presented in Table 6. The histograms reported in Figure 1 showed that essential oils reduce cell viability in a dose- and time-dependent manner. The results indicate that the essential oils from the leaves and stems of *A. indica* have promising cytotoxic capabilities against MCF-7 cells, with IC_50_ values of 10.04 ± 1.82 and 15.89 ± 1.66 μg/mL after 72 h of treatment, respectively. Interestingly, the leaf EOs exhibited more cytotoxicity against all the cell lines tested compared to the stem EOs. However, both LEOs and SEOs exhibited weaker growth inhibitory activity against cell lines compared to doxorubicin. The selectivity indices (SIs) were determined by taking the ratio of the IC_50_ values (48 h) of LO2, representing non-cancerous cells, to the IC_50_ values (48 h) of the cancer cells. The selectivity index of EOs was calculated in the range of 0.95 to 3.38 for LEOs and 0.80 to 1.72 for SEOs. The LEOs presented the best selectivity index (3.38) against the MCF-7 cell lines.

## 3. Discussion

Differences in the constituents of the leaf and stem EOs derived from *A. indica* have been observed. The predominant compounds in the LEOs and SEOs were linalool (4.1% and 11.8%), (*E*)-caryophyllene (17.3% and 10.8%), *α*-guaiene (4.7% and 3.1%), viridiflorol (8.1% and 9.5%), etc. *trans*-Verbenol was detected in LEOs at a concentration of 4.9%; however, it was not detected in SEOs. In SEOs, hexadecanoic acid accounted for 6.3% of the total, whereas in LEOs, it was less than 1%. The essential oil constituents of other legume plants have been reported to be rich in similar compounds, such as (*E*)-caryophyllene [22,23,24,25,26], phytol [22,26], (*E*)-nerolidol [27], pentadecanal [27], and δ-cadinene [24]. However, cis-verbenol and trans-verbenol have not been reported.

Recent research demonstrated that the major compounds observed in *A. indica* EOs possess potent bioactive capabilities. For example, (*E*)-caryophyllene, which was found in both SEOs and LEOs, is well-known for its antibacterial [28,29], antifungal [29], anti-inflammatory [30], and anticancer activities [29,31]. Furthermore, viridiflorol demonstrated strong anticancer activity against the Daoy cells and MCF-7 [32] and is widely utilized as an antioxidant, anti-tuberculosis, and anti-inflammatory agent [33]. Linalool has also been found to exhibit anti-inflammatory [34,35], antimicrobial [36,37,38,39], anticancer [40], and neuroprotective activities [41].

Bacteria are responsible for a number of detrimental effects on human health, the deterioration of food products, and a multitude of other issues. With the emergence of drug-resistant bacteria, there is a necessity to explore alternative sources of defense against pathogenic bacteria. EOs and their components play a pivotal role in inhibiting the growth of microorganisms [42]. It is well established that essential oils possess antibacterial activities, particularly against Gram-positive bacteria [43,44], such as *Staphylococcus aureus*. This bacterium is a significant pathogen responsible for various human illnesses, ranging from mild infections of skin and soft tissue to severe tissue and sepsis [45,46]. The LEOs and SEOs of *A. indica* showed inhibitory effects against both Gram-positive and -negative bacterial strains that were examined in this study. Compared to the stem essential oils, the LEOs showed higher activity against *S. aureus* and *P. aeruginosa*, with MICs of 0.312 and 1.250 mg/mL for LEOs and 0.625 and 2.500 mg/mL for SEOs, respectively. In addition, the essential oils exhibited similar antibacterial activities against *B. subtilis* and *E. coli*, with the same MIC values of 0.312 and 1.250 mg/mL, respectively. The variations revealed in the compositions of the two essential oils may explain their varying levels of biological activity in the current investigation [47]. The presence of bioactive compounds, including (*E*)-caryophyllene, viridiflorol, and linalool, was responsible for this activity; these compounds have been reported to possess antibacterial potential [29,48,49,50,51,52]. In contrast to tested Gram-positive bacterial strains, the oils exhibited lower efficacy against Gram-negative bacteria. The discrepancies in antibacterial efficacy may be attributed to the net repulsion of the two outer complex membranes’ structure in the Gram-negative bacterial cell wall, which is absent in Gram-positive bacteria [53]. Essential oils exert biological activities through a variety of mechanisms due to their diversity in chemical composition [54]. The antibacterial action of essential oils is multifaceted and consists of a combination of disrupting cell membranes, inducing oxidative stress, damaging genetic material, and inhibiting enzyme activity [54]. Among these, the most significant mechanism of action is the disruption of bacterial cell membranes. Essential oils’ lipophilic character makes them easily penetrable through bacterial cell membranes, which breaks membrane integrity and increases permeability, causing disruption to many cellular activities, including energy production (membrane-coupled), membrane transport, and other metabolic regulating functions [55].

Recently, the combination of conventional antibiotics with medicinal natural products has become a popular strategy to reverse the antibacterial activity of failed antibiotics in treating infections that are resistant to these drugs via drug–drug interactions involving the action of multiple antibacterial mechanisms. These strategies may prevent the emergence of novel resistance mechanisms in bacteria, minimize the use of antibiotics while maintaining current antibiotic classes for therapeutic benefits, and mitigate adverse effects [56]. The current investigation demonstrated that the combination of *A. indica* EOs and antibiotics possessed a synergistic interaction in all examined bacteria, as indicated by an FIC index value of less than 0.5. Moreover, the concentration of essential oils and antibiotics in the combined test was much lower than that of the single chemical concentration. The maximum synergistic effect was observed for *S. aureus* and *P. aeruginosa*, with a 16-fold gain compared to streptomycin’s MIC. It is noteworthy that chloramphenicol or streptomycin, in combination with EOs extracted from different parts of *A. indica*, showed a strong synergistic effect against both Gram-positive and Gram-negative bacteria tested. This observation suggests that *A. indica* essential oils may serve as promising effective natural adjuvant agents in conjunction with traditional antibiotics, with the aim of reducing the risk of developing antibiotic-resistant microorganisms.

The in vitro antioxidant activities of *A. indica* LEOs and SEOs were evaluated in three different models, including DPPH, ABTS, and FRAP. Overall, in the three tests, both LEOs and SEOs exhibited antiradical capacities against different oxidants and displayed moderate ferric reducing abilities. The differences noticed in the activities of the leaves and stem EOs towards the two distinct radicals (DPPH^•^ and ABTS^•+^) can be assigned to several factors, such as the complications, polarity, and selectivity of isomers of the radicals [57]. Scavenging of ABTS^•+^ by the EOs from different parts of *A. indica* was higher than that of DPPH^•^ radicals. This may be because ABTS^•+^ cation radicals are more sensitive to high-molecular-weight phenolics and more reactive than DPPH^•^ radicals, wherein the reaction of ABTS^•+^ radicals with antioxidants is completed within 1 min [58]. Furthermore, the solvation ability of the oils towards the radical’s medium may vary, and these factors have been found to impact the effectiveness of volatile components in suppressing various types of radicals [57]. The high content of phytol in the essential oils in the present study is noteworthy and likely contributed to the improved antioxidant properties of these EOs. Phytol, a diterpenoid alcohol, has demonstrated strong in vitro antioxidant activity, effectively eliminating hydroxyl radicals and preventing the development of thiobarbituric acid reactive substances [59].

The utilization of medicinal plant chemicals for the development and treatment of a multitude of diseases, especially cancer, has been researched for decades [60]. In this study, we examined the in vitro cytotoxic effects of EOs and determined the IC_50_ values on A-549, MCF-7, HepG2, and HCT-116 cells. We present evidence demonstrating the cytotoxic effects of both LEOs and SEOs in tested cell lines of diverse origins. The data indicated that the MCF-7 cell line exhibited a more favorable response to *A. indica* EOs, demonstrating enhanced cytotoxicity. When MCF-7 cells were exposed to *A. indica* essential oils, a notable potential for cytotoxic effects was identified, which indicates that *A. indica* essential oils could be a promising therapy option for patients with breast cancer. Early research indicates that (*E*)-caryophyllene exhibits significant cytotoxic activity against various cancer cells, including A-549 (lung carcinoma), HeLa (cervical carcinoma), and HT-29 (colon adenocarcinoma) cells [29,31]. Additionally, it has been shown to potentiate the effects of conventional chemotherapy agents such as doxorubicin and paclitaxel, indicating its potential as an adjunct in chemotherapeutic regimens [61]. Furthermore, studies have demonstrated the effectiveness of viridiflorol in inducing apoptosis in different cell lines, including A549, Daoy, and MCF-7 [32].

The additive or synergetic effects of the bioactive compounds identified in the EOs may be responsible for the greater biological activity of the LEOs in comparison to the SEOs. The biological activities of the LEOs may have been enhanced by other terpenoid constituents, even in small amounts, for example, by *β*-pinene, limonene, and *β*-elemene, which have previously been reported to possess remarkable bioactivities [62,63,64,65,66,67,68].

## 4. Materials and Methods

### 4.1. Plant Material

*A. indica* leaves and stems were obtained from Quzhou, located in Zhejiang Province, China, in September 2022. Dr. Hong Zhao, Shandong University, China, verified the classification of the plant and placed a voucher specimen (No. 022021) in the herbarium of the Department of Biological Sciences at Shandong University in Weihai, China.

### 4.2. Essential Oil Extraction

The plant materials (1 kg of leaves or stems of *A. indica*) were hydrodistilled for three hours using a Clevenger-type device to derive EOs and then kept at 4 °C for analysis. The experiments were conducted three times.

### 4.3. Identification of EO Components

GC and GC/MS were used to analyze the composition of EOs. Agilent Technologies 7890A (St. Clara, CA, USA) gas chromatography equipped with FID and a non-polar HP-5MS column, using helium as the carrier at a flow rate of 1.3 mL/min, was used to perform the GC analysis. The GC column oven was set from 60 °C (held for 1 min) to 250 °C (held for 14 min), with an increase of 8 °C/min. The injector temperature was 260 °C. The EOs were diluted with hexane to 1% (*v*/*v*) and injected into the GC system. Mass spectra were recorded at 70 eV. The mass scanning range was from 50 to 550 *m*/*z*.

The identification of LEO and SEO components was conducted by comparing their mass spectra to those in the NIST and WILEY libraries and by comparing their retention indices (retention indices relative to C_10_–C_30_ n-alkanes) to the standard RI provided in the literature [69,70,71].

### 4.4. Antibacterial Activity Assays

The method of microdilution of the broth was employed to assess the antibacterial potential of EOs [72]. The strains examined were *P. aeruginosa*, *E. coli*, *B. subtilis*, and *S. aureus*. EOs were diluted in 96-well microplates using a two-fold serial dilution method, with 100 μL added to each well. Subsequently, the dilutions were combined with a bacterial solution consisting of 100 μL with a concentration of 10^6^ CFU/mL. The positive control utilized was chloramphenicol. After a 24-h incubation at 37 °C, the growth of the bacteria was evaluated using TTC as a growth indicator. The MIC of the sample was the lowest concentration with no bacterial growth. The MBC was identified by inoculating 100 μL of subculture from the well, with no visible growth of bacteria occurring after incubation at 37 °C for 24 h.

### 4.5. Synergistic Antibacterial Activity of EOs

The synergistic effects of the combination of chloramphenicol or streptomycin with EOs were assessed using the micro broth checkboard method [73]. Sample concentrations varied from 4 to 1/32 times the MIC, depending on previously determined MIC values. In summary, 50 μL of LEOs or SEOs was introduced at two-fold dilution concentrations into different microwells of the 96-well plates. These microwells already contained 50 µL of various antibiotic concentrations and 100 μL of bacterial suspension (10^6^ CFU/mL). After incubation at 37 °C for 24 h, the MIC of EO or antibiotics tested alone and in combination were determined. The FICI value was calculated as a predictor of synergy by using the following formula:

(1) $$\mathrm{FICI}=\frac{\mathrm{MIC}\;\mathrm{of}\;\mathrm{EO}\;\mathrm{in}\;\mathrm{combination}}{\mathrm{MIC}\;\mathrm{of}\;\mathrm{EO}\;\mathrm{alone}}+\frac{\mathrm{MIC}\;\mathrm{of}\;\mathrm{antibiotic}\;\mathrm{in}\;\mathrm{combination}}{\mathrm{MIC}\;\mathrm{of}\;\mathrm{antibiotic}\;\mathrm{alone}}$$

Synergy is considered to exist if FICI ≤ 0.5 [74].

### 4.6. Antioxidant Activity Evaluation

#### 4.6.1. DPPH Radical Scavenging

The DPPH test was conducted by the 96-well plate method, as described in reference [75]. Briefly, 150 μL of a 0.05 mg/mL DPPH solution was added to each well of a plate. Additionally, 50 μL of the sample at varying concentrations were added to the wells, excluding the blank test wells. The control test well received an addition of 200 µL of methanol. The solutions were shaken for 1 min and subsequently placed in darkness for 6 h. The absorbance was quantified at 517 nm. The standard reference compounds utilized were BHT and Trolox. Radical inhibition (I%) was calculated as follows:

I (%) = [1 − (A_sample_ − A_blank_)/A_blank_)] × 100

A represents absorbance.

#### 4.6.2. ABTS Radical Scavenging

The ABTS test was performed according to a previously published procedure with minor adjustments [75]. Briefly, 7 mM ABTS was mixed with 2.45 mM potassium persulfate to give an ABTS^•+^ solution, which was left in the darkness for 16 h. The ABTS^•+^ solution was diluted in PBS to an absorbance of about 0.700 (±0.02) at a wavelength of 734 nm. The EOs were mixed with methanol to obtain the desired concentrations. Afterward, 50µL of the sample solution was combined with 150 µL of the ABTS^•+^ solution in a 96-well plate. The mixture was placed in a lightless environment for 30 min, after which the absorbance was quantified at 734 nm. The ABTS^•+^ scavenging effect was determined using the same formula that was used for DPPH.

#### 4.6.3. Ferric Reducing Power

The ferric reducing power was assayed using a previous method with minor modifications [75]. The FRAP reagent included 300 mM acetate buffer (pH 3.6), 10 mM TPTZ solution in 40 mM HCl, and FeCl_3_·6H_2_O (20 mM) in a proportion of 10:1:1. Afterwards, 20 μL serial dilutions of EOs and 180 μL of the FRAP reagent were combined in a 96-well plate in darkness and then incubated at 37 °C for 30 min. The absorbance was measured at 593 nm. The activity was quantified as the Trolox-equivalent antioxidant capacity.

### 4.7. Cytotoxic Activity Evaluation

The cytotoxic effects of EOs on four different types of cancer cells (MCF-7, A-549, HCT-116, and HepG2), as well as non-cancer LO2 cells, were evaluated by an MTT assay, as previously reported [76]. The tested cell lines were obtained from the Shanghai Institute for Biological Sciences (SIBS, Shanghai, China). The cells were cultured in an RPMI 1640 medium supplemented with 10% fetal bovine serum, 2 mM glutamine, 100 units/mL penicillin, and 100 μnits/mL streptomycin at 37 °C with a 5% CO_2_. A total of 5 × 10^4^ cells were distributed into each well of a 96-well plate, along with 100 μL of an RPMI medium and 10% FBS. Following incubation for 24 h, the EOs were diluted in a two-fold serial dilution to a concentration of 6.25 to 400.00 μg/mL and then added to each well. After 48 h, 20 μL of MTT solution (5 mg/mL) was added to each well. The plates were subsequently incubated for an additional 4 h. The formazan blue produced in the cell was dissolved in 150 μL of DMSO solution. The absorbance was quantified at 570 nm.

### 4.8. Statistical Analysis

All the experiments were carried out in triplicate, and the data were presented as the mean ± SD. Statistical analysis of the data was carried out using the SPSS software package (IBM SPSS Statistics, v. 29). One-way ANOVA was used to test the statistical significance of the data. Differences were considered statistically significant at a significance level of *p* ≤ 0.05.

## 5. Conclusions

This study evaluated the composition and antioxidant, antibacterial, synergistic antibacterial, and cytotoxicity effects of EOs. The primary constituents of EOs included (*E*)-caryophyllene, linalool, viridiflorol, phytol, hexadecanoic acid, trans-verbenol, and α-guaiene, among others. By utilizing three distinct methodologies to assess antioxidant activity, the essential oils demonstrate a promising capacity to eliminate ABTS radicals. In addition, the essential oils of *A. indica* showed stronger inhibitory effects against *S. aureus* compared to other types of microorganisms. Furthermore, a synergistic impact was detected when essential oils were combined with antibiotics, resulting in the effective inhibition of all tested bacterial strains. MTT assays demonstrated the cytotoxic effects of both LEOs and SEOs in tested cell lines of diverse origins, especially MCF-7 cells. These findings indicate that these oils possess the capacity to serve as a natural antibacterial agent, offering prospective applications in the food, pharmaceutical, and cosmetics sectors.

## Figures and Tables

**Table 1:** Chemical composition of LEOs and SEOs of *A. indica*.

Peak No.	Compound	RI ^a^	RI ^b^	% (Leaves)	% (Stems)
1	*β*-Pinene	975	980	2.2	-
2	Limonene	1028	1029	1.0	-
3	*p*-Cymenene	1089	1089	0.6	0.6
4	Linalool	1099	1095	4.1	11.8
5	*α*-Campholenal	1126	1122	1.3	0.6
6	*cis*-Limonene oxide	1132	1132	-	1.8
7	*trans*-Pinocarveol	1140	1139	-	0.6
8	*cis*-Verbenol	1141	1141	0.9	-
9	*trans*-Verbenol	1145	1144	4.9	-
10	1,4-Dimethyl-4-acetylcyclohexene	1151	1152	-	1.2
11	Pinocarvone	1163	1164	1.4	1.2
12	Terpinen-4-ol	1178	1177	0.7	1.0
13	*p*-Cymen-8-ol	1185	1182	0.5	0.8
14	*α*-Terpineol	1191	1189	0.8	3.1
15	Myrtenol	1197	1195	2.3	1.3
16	*n*-Decanal	1203	1201	-	1.2
17	Verbenone	1210	1205	0.9	-
18	*β*-Cyclocitral	1222	1219	1.4	1.6
19	Nerol	1228	1229	-	1.2
20	Geraniol	1253	1252	0.7	1.9
21	*β*-Cyclohomocitral	1259	1261	0.2	-
22	(*E*,*E*)-2,4-Decadienal	1315	1315	-	0.7
23	Dehydro-ar-ionene	1355	1355	0.3	0.4
24	Eugenol	1358	1359	0.5	-
25	Copaene	1378	1376	2.3	1.0
26	*β*-Damascenone	1385	1384	1.3	1.3
27	*β*-Elemene	1393	1391	1.1	0.7
28	(*Z*)-Caryophyllene	1410	1408	0.4	0.2
29	*α*-*trans*-Bergamotene	1417	1432	0.6	-
30	(*E*)-Caryophyllene	1424	1419	17.3	10.8
31	(*E*)-*α*-Ionone	1434	1434	0.5	-
32	*α*-Guaiene	1441	1439	4.7	3.1
33	Geranyl acetone	1451	1453	0.4	0.8
34	Humulene	1458	1454	1.4	0.9
35	2,4,6-Trimethoxy-toluene	1471	1482	1.0	0.3
36	*β*-Chamigrene	1478	1477	2.2	1.2
37	(*E*)-*β*-Ionone	1487	1487	2.5	3.3
38	*β*-Selinene	1492	1498	0.7	0.5
39	Aciphyllene	1501	1501	-	0.3
40	*α*-Bulnesene	1508	1509	1.2	0.8
41	*δ*-Cadinene	1526	1524	2.0	0.9
42	*α*-Calacorene	1547	1544	0.5	-
43	Diepicedrene-1-oxide	1557	1551	0.5	0.4
44	(*E*)-Nerolidol	1563	1563	1.3	2.0
45	(3*Z*)-Hexenyl benzoate	1571	1566	0.8	0.7
46	Caryophyllene oxide	1578	1582	-	0.4
47	Viridiflorol	1589	1592	8.1	9.5
48	Tetradecanal	1609	1611	-	0.4
49	Humulene epoxide II	1610	1608	0.6	-
50	Junenol	1614	1618	0.5	0.5
51	Caryophylladienol II	1641	1639	0.3	0.4
52	Zizanol	1663	1677	0.4	0.5
53	Bulnesol	1676	1671	1.4	1.1
54	Cryptofauronol	1692	1713	0.3	0.3
55	Pentadecanal	1711	1715	3.4	1.6
56	Tetradecanoic acid	1758	1758	-	0.6
57	Benzyl Benzoate	1767	1760	0.3	0.7
58	(*E*)-Isovalencenol	1791	1793	0.4	-
59	*β*-Costol	1792	1788	-	0.6
60	Hexahydrofarnesyl acetone	1841	1843	1.9	1.8
61	*n*-Hexadecanol	1876	1874	0.3	-
62	(5*E*,9*E*)-Farnesyl acetone	1915	1913	0.5	1.1
63	Methyl palmitate	1921	1921	-	0.6
64	Isophytol	1944	1946	0.1	0.4
65	Hexadecanoic acid	1956	1959	0.2	6.3
66	*n*-Octadecanol	2079	2077	3.5	2.9
67	Phytol	2109	1943	5.2	7.0
68	Oleic Acid	2139	2141	0.3	0.7
	Monoterpene hydrocarbons			4.1	1.0
	Oxygenated monoterpenes			21.0	32.9
	Sesquiterpene hydrocarbons			34.4	20.4
	Oxygenated sesquiterpenes			16.2	18.6
	Oxygenated diterpenes			5.3	7.4
	Total identified			95.1	97.6

^a^ Retention index calculated from n-alkanes (C_7_–C_30_) on an HP-5MS column. ^b^ Retention index data from the literature.

**Table 2:** Antibacterial activities of LEOs and SEOs of *A. indica*.

Strain	MIC (mg/mL)			MBC (mg/mL)		
	LEOs	SEOs	Ch	LEOs	SEOs	Ch
Gram-positive						
*B. subtilis* ATCC 6633	0.312	0.312	0.004	0.312	0.625	0.008
*S. aureus* ATCC 6538	0.312	0.625	0.004	0.312	0.625	0.016
Gram-negative						
*E. coli* ATCC 25922	1.250	1.250	0.008	2.500	2.500	0.031
*P. aeruginosa* ATCC 27853	1.250	2.500	0.063	2.500	2.500	0.250

Ch: Chloramphenicol.

**Table 3:** Effect of the combination of EOs with chloramphenicol.

Strain	Sample	MICa (μg/mL)	MICc (μg/mL)	FICI
*Bacillus subtilis*	LEOs	312.50	9.77	0.28 (S)
	Chl	4.00	1.00	
	SEOs	312.50	78.13	0.50 (S)
	Chl	4.00	1.00	
*Staphylococcus aureus*	LEOs	312.50	19.53	0.19 (S)
	Chl	4.00	0.50	
	SEOs	625.00	78.13	0.38 (S)
	Chl	4.00	1.00	
*Escherichia coli*	LEOs	1250.00	156.25	0.25 (S)
	Chl	8.00	1.00	
	SEOs	1250.00	312.50	0.38 (S)
	Chl	8.00	1.00	
*Pseudomonas aeruginosa*	LEOs	1250.00	78.13	0.19 (S)
	Chl	62.50	8.00	
	SEOs	2500.00	156.25	0.32 (S)
	Chl	62.50	16.00	

MICa: MIC alone; MICc: MIC combined; and Chl: chloramphenicol. S: synergy.

**Table 4:** Effect of the combination of EOs with streptomycin.

Strain	Sample	MICa (μg/mL)	MICc (μg/mL)	FICI
*Bacillus subtilis*	LEOs	312.50	19.53	0.19 (S)
	SM	2.00	0.25	
	SEOs	312.50	9.77	0.16 (S)
	SM	2.00	0.25	
*Staphylococcus aureus*	LEOs	312.50	9.77	0.10 (S)
	SM	2.00	0.13	
	SEOs	625.00	39.10	0.19 (S)
	SM	2.00	0.25	
*Escherichia coli*	LEOs	1250.00	39.10	0.16 (S)
	SM	4.00	0.50	
	SEOs	1250.00	312.50	0.38 (S)
	SM	4.00	0.50	
*Pseudomonas aeruginosa*	LEOs	1250.00	78.13	0.13 (S)
	SM	4.00	0.25	
	SEOs	2500.00	156.25	0.19 (S)
	SM	4.00	0.50	

MICa: MIC alone; MICc: MIC combined; SM: streptomycin. S, synergy.

**Table 5:** Results of the antioxidant activity (DPPH, ABTS, and FRAP) of the EOs from different parts of *A. indica*.

Test Sample	DPPH IC_50_ (mg/mL) ^a^	ABTS IC_50_ (mg/mL) ^a^	FRAP (μmol Trolox × g^−1^)
LEOs	1.35 ± 0.06 ^a^	0.11 ± 0.01 ^a^	103.78 ± 10.23 ^a^
SEOs	1.66 ± 0.05 ^a^	0.19 ± 0.01 ^b^	57.75 ± 7.63 ^b^
BHT *	(8.00 ± 0.38) × 10^−3 b^	(6.00 ± 0.38) × 10^−3 c^	
Trolox *	(6.00 ± 0.46) × 10^−3 c^	(4.00 ± 0.23) × 10^−3 d^	

* Positive control used. The different superscript letters within a column show statistically significant differences (*p*  <  0.05).

**Table 6:** Cytotoxicity (IC_50_, μg/mL) of the LEOs and SEOs of *A. indica*.

	Sample	24 h	48 h	72 h
HepG2	LEOs	60.37 ± 2.17 ^a^	36.05 ± 4.89 ^a^	23.90 ± 2.00 ^a^
	SEOs	74.09 ± 8.17 ^b^	51.91 ± 2.54 ^b^	34.82 ± 3.97 ^b^
	Doxorubicin	2.16 ± 0.16 ^c^	1.68 ± 0.08 ^c^	1.43 ± 0.07 ^c^
MCF-7	LEOs	40.83 ± 4.48 ^a^	11.92 ± 1.97 ^a^	10.04 ± 1.21 ^a^
	SEOs	51.30 ± 3.58 ^b^	25.01 ± 0.98 ^b^	15.89 ± 0.30 ^b^
	Doxorubicin	1.56 ± 0.03 ^c^	1.34 ± 0.05 ^c^	1.19 ± 0.08 ^c^
LO2	LEOs	50.28 ± 0.61 ^a^	40.32 ± 1.53 ^a^	31.33 ± 0.73 ^a^
	SEOs	64.31 ± 2.45 ^b^	43.12 ± 1.18 ^b^	38.73 ± 3.72 ^b^
	Doxorubicin	1.80 ± 0.29 ^c^	0.82 ± 0.04 ^c^	0.55 ± 0.12 ^c^
A-549	LEOs	57.60 ± 4.85 ^a^	42.52 ± 2.58 ^a^	33.19 ± 2.85 ^a^
	SEOs	68.18 ± 6.47 ^a^	53.76 ± 5.15 ^b^	48.66 ± 6.54 ^b^
	Doxorubicin	1.64 ± 0.07 ^b^	1.41 ± 0.11 ^c^	0.93 ± 0.01 ^c^
HCT-116	LEOs	46.82 ± 5.45 ^a^	35.54 ± 2.36 ^a^	33.81 ± 1.00 ^a^
	SEOs	67.38 ± 2.98 ^b^	53.76 ± 7.30 ^b^	44.92 ± 4.08 ^b^
	Doxorubicin	1.33 ± 0.16 ^c^	0.95 ± 0.05 ^c^	0.88 ± 0.06 ^c^

IC_50_: the concentration of the compound that affords a 50% reduction in cell growth (after 24, 48, and 72 h of incubation)—expressed as the mean ± SD of triplicate experiments. The mean values with different letters within a column in different cell lines are significantly different according to Tukey’s test (*p*  <  0.05).

## Data Availability

The data are contained in this article.
